# Different Development Forms of Local Allergic Rhinitis towards Birch

**DOI:** 10.1155/2020/3408561

**Published:** 2020-06-04

**Authors:** Andrzej Bozek, Janne Winterstein, Beata Galuszka, Jerzy Jarzab

**Affiliations:** ^1^Clinical Department of Internal Medicine, Dermatology and Allergology, Medical University of Silesia, Katowice, Poland; ^2^Allergy Outpatient Clinic, Research Department, Munchen, Germany; ^3^PMR Allergy Department, Outpatient Clinic, Swietochlowice, Poland

## Abstract

**Background:**

Efficacy of allergen immunotherapy (AIT) in local allergic rhinitis (LAR) is a new subject of research. The presence of asthmatic symptoms in patients with LAR in the context of AIT is unexplored.

**Objective:**

The efficacy and safety of AIT in patients with LAR towards birch pollen were investigated. The possibility of concomitant local allergic asthma in studied patients and the impact of AIT on it were examined.

**Methods:**

36 patients with LAR towards birch were included in three years of AIT in a double-blind, placebo-control study. Primary outcome measurement was the mean changes in the combined symptom and medication scores (CSMSs) after AIT, and the second is the changes in the quality of life (QoL). Skin prick tests, serum, nasal allergen-specific IgE to birch, nasal and bronchial provocation challenge tests with birch allergen, methacholine tests, and spirometry were carried out at baseline and after AIT.

**Results:**

Mean CSMSs of three years of AIT were significantly decreased in the active group from 5.88 (range: 4.11-9.01) to 1.98 (range: 1.22-4.51; *p* < 0.05). After three years of AIT, there was a significant increase of toleration for birch allergen from the mean concentration of 6250 ± 1200 SQ-U/ml up to 45000 ± 2500 SQ-U/ml (*p* = 0.02) during repeated nasal challenges. 16 patients with LAR had the positive results of methacholine tests, and 11 of them had a positive bronchial challenge to birch allergen. After AIT, the significant decrease of bronchial responsiveness to birch allergen in 5 from 7 patients was confirmed (*p* = 0.03). QoL assessed by the use of the RQLQ score was improved after AIT from 1.84 (95% CI: 1.53-1.97) to 1.45 (95% CI: 1.32-1.62) score in the active group after three years of AIT therapy (*p* = 0.03).

**Conclusion:**

AIT to birch can be useful and safe in a patient with local allergic rhinitis and also with concomitant asthmatic symptoms. Further studies are needed.

## 1. Background

Allergic rhinitis and allergic asthma are common diseases that are well diagnosed and effectively treated [1, 2]. Many guidelines and position papers have presented the epidemiology, causes, pathomechanism, and management of these diseases [3–5]. Nowadays, in terms of precision medicine, many different endotypes of rhinitis and asthma are still under investigation [6]. This leads to a better understanding of the mechanism of the disease and the recommendation of different treatments for a different type of the same diseases. An example of this is the use of biological drugs in severe asthma with eosinophilia or allergen immunotherapy in some allergic rhinitis [7, 8].

Unfortunately, there are still many forms of allergic rhinitis and asthma that could not be easily determined. Recently, local allergic rhinitis (LAR) has been defined and presented as one of the endotypes [9]. The diagnosis and treatment of this form of rhinitis are still of great interest. The lack of evidence for a systemic allergic reaction does not preclude a similar local response in the nasal mucosa to a specific allergen [10]. Confirmation of this reaction through nasal provocation tests enabled the appropriate treatment of such patients. However, there is still little scientific evidence of the effectiveness of allergen immunotherapy in patients with local allergic rhinitis [11, 12]. It also seems that some of these patients may present symptoms of bronchial asthma, despite attempts to confirm it in additional tests.

Can the phenomenon of local allergic reaction be present in the bronchi as in the nose?

Based on these questions and doubts, the authors put forward the following hypotheses:

1. Do patients with confirmed LAR towards birch have relief of symptoms after allergen immunotherapy?
2. Do these patients also have asthmatic symptoms towards birch despite negative skin prick test and IgE?

## 2. Methods

### 2.1. Study Design

It was a prospective, double-blind, placebo-controlled, multicenter study conducted with patients confirmed with LAR towards birch pollen.

### 2.2. Patients

First, we prescreened approximately 102 patients 18-77 years old who had clinical symptoms of allergy in birch pollen season, completely negative skin tests, and specific IgE to inhalant allergens and suspicion of LAR.

All patients were recruited based on the following criteria:

1. >18 yrs old
2. Well-documented symptoms of mild, moderate, or severe intermittent rhinitis according to the Allergic Rhinitis and its Impact on Asthma (ARIA) during birch pollen season [1]
3. A positive nasal provocation test to birch
4. Negative skin prick test results for common inhalant allergens, including *D. pteronyssinus*, *D. farinae*, grass pollen, birch, hazel, alder, *Alternaria*, and cats
5. Negative serum total and allergen-specific IgE results against the mentioned allergens
6. Provided consent to participate in the study

Other clinical symptoms such as cough and/or dyspnea during the birch pollen season did not exclude patients from the study.

**Figure 1:**
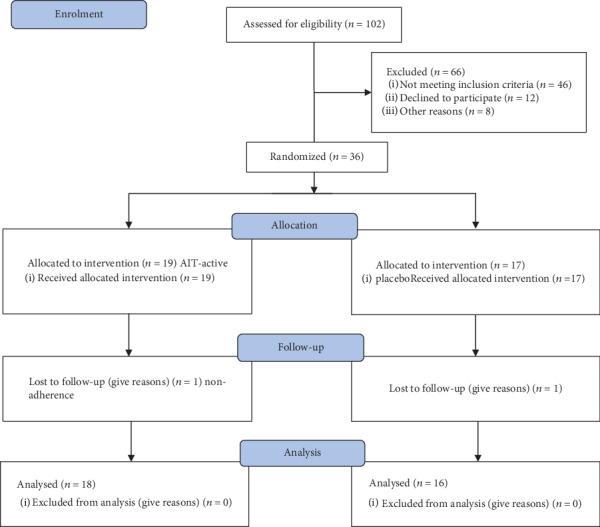
The number of participants assessed for eligibility that completed the study.

The exclusion criteria were as follows: clinical exacerbation of chronic rhinosinusitis or respiratory infections within 4 weeks prior to the study initiation; nasal polyposis (which is contraindicated for nasal provocation) or other serious diseases or chronic unstable disease; and nose deformity, allergy to other inhalant allergens, and systemic allergic rhinitis. All patients with any of the following characteristics were also excluded: diagnosis of chronic bronchial asthma, nonallergic rhinitis (especially senile or vasomotor rhinitis), and severe nonstable diseases. All subjects were required to abstain from antiallergy drugs and glucocorticoid nasal drops for at least 6 weeks prior to the start of the study. The process of including patients is presented in Figure 1.

### 2.3. Study Protocol

Patients who met the inclusion criteria were selected to undergo further procedures as follows at baseline: medical examinations, skin prick tests with inhalant allergens, serum total IgE and specific IgE antibody levels, bronchial reversibility test, nasal and bronchial challenges with birch allergen, and methacholine test.

### 2.4. Rhinitis Severity

The severity of ocular and nasal symptoms, including obstruction, rhinorrhoea (watery, mucous, and purulent), itching, and sneezing, was recorded using a visual analogue scale (VAS) of 10 cm. Each symptom was categorized as “mild” (VAS: 0–30 cm), “moderate” (VAS: >30 cm and ≤70 cm), or “severe” (VAS: >70 cm) [1].

### 2.5. Examination

A full rhinolaryngological examination was performed using anterior and posterior rhinoscopy, and in some patients, endoscopy and CT scan were performed.

Rhinitis was classified according to the following Allergic Rhinitis and its Impact on Asthma (ARIA) guidelines: rhinitis is considered persistent when symptoms are present for >4 days/week or persist for >4 consecutive weeks. The rhinitis severity was based on estimations of activity impairment (sleep, daily activities, work/school performance, and troublesome behaviour) and was classified as severe, moderate, or mild [1].

If there were appropriate clinical symptoms, asthma was diagnosed based on the GINA criteria [2].

### 2.6. Skin Prick Test (SPT)

The SPT was performed using a panel of the following aeroallergens: birch, *D. pteronyssinus*, *D. farinae*, *Phleum pratense*, *Artemisia*, alder, hazel, *Alternaria*, *Cladosporium*, *Aspergillus*, and cockroach, dog, and cat epithelia (Soluprick SQ, ALK-Abello, Horsholm, Denmark). A positive control (10 mg/ml histamine) and negative control (saline) were included. An allergic reaction was defined as a positive skin test for at least one allergen, with a maximum wheal diameter of at least 3 mm greater than that of the negative control. Patients who did not exhibit a reaction to histamine were excluded from further analyses [13].

### 2.7. Serum-Specific IgE (sIgE) and Serum-Specific IgG_4_ to Bet v1

Serum total and sIgE antibody levels to the same aeroallergens as used in the SPT panel were determined using a fluoroenzyme immunosorbent assay (Thermo Fisher, Uppsala, Sweden). The positive cutoff value for sIgE levels was >0.35 kU/l. Separately, IgE and IgG_4_ to Bet v1 were performed in baseline and after therapy.

### 2.8. Nasal Provocation Test (NPT)

Nasal provocation tests were performed using acoustic rhinometry with an Acoustic Rhinometer A1 (GM Instruments, Kilwinning, UK). These tests were performed according to the guidelines of the Standardization Committee on Acoustic Rhinometry and the EAACI position paper [14, 15]. The nasal provocation tests were performed when the concentrations of the examined allergens were lower in Poland. First, using a metered pump spray, the patients were intranasally challenged with saline to exclude nasal hyperreactivity. If the nasal provocation test was negative, it was performed again one week later with another provocation with saline as the negative control and then with the NPT with extracts of birch: 1000, 10000, and 100 000 SQ (Aquagen, ALK -Abello). A total of one hundred microliters of the solution of allergen was applied to each nostril. The total volume of both nasal cavities was determined to be 2-6 cm using acoustic rhinometry, and the results were compared with the baseline test. The immediate reaction was analyzed at 15 min, 1 hour, and 6 hours according to the protocol based on the EAACI position paper [14].

### 2.9. Nasal-Specific IgE (nsIgE) Detection

The nsIgE levels to the same aeroallergens were examined identically to serum sIgE by the use of immunoassay and were presented in kU/L (Thermo Fisher, Uppsala, Sweden). According to the manufacturer, the cutoff for positive result was >0.35 kU/L. These measurements were performed at baseline, immediately (30 sec) after allergen provocation, and at 15 minutes, 1 hour, and 6 hours after the nasal positive provocation tests.

A bilateral nasal lavage was performed according to Nacleiro et al. [16]. All the values are provided as a calculated protein ratio.

### 2.10. Diagnosis of LAR

Patients have confirmed local allergic rhinitis if there were positive NPT to birch, positive detection of nsIgE to birch and negative SPT, and sIgE to all common inhalant allergens including birch.

### 2.11. Spirometry

Spirometry was performed outside the pollen season. Pulmonary function tests, in addition to baseline spirometry and a reversibility bronchial test at the first visit, were performed according to the standards of GINA. The test was positive according to GINA guidelines if there was an improvement in FEV1 ≥ 12% together with an increase in volume ≥ 200 ml [2].

### 2.12. Methacholine Test and Bronchial Challenge

The bronchial provocation test was carried out using DeVilbiss 464 nebulizer and Jaeger system (Jaeger, Hoechberg, Germany). The bronchial challenge with the methacholine test was performed by the use of the body plethysmography Jaeger Vyntus APS (Jaeger, Hoechberg, Germany). Patients received a cumulative dose of 31-215 *μ*g using a standard dilution of 3.3 mg/100 ml. A methacholine test was done one week before allergen provocation [17].

The bronchial challenge with birch allergen extract Aquagen (ALK-Abello, Horsholm, Denmark) was administered from negative diluent, and then, the five doses were used every 30 minutes as follows: 1000 SQ,10 000 SQ, and 100 000 SQ. Spirometry was performed after 20 minutes of applying steps of allergen inhalation. The time interval between consecutive steps was 30 min.

For each challenge, it was finished if FEV1 decrease by 20% from baseline measurement and it was confirmed as positive results. Patients were observed 24 hours after bronchial challenge.

All procedures were performed in October-November and after three years of therapy in October-November.

### 2.13. Intervention

#### 2.13.1. Randomization

The thirty-six patients were randomized (1 : 1) for the administration of perennial AIT with Alutard SQ *Betula verrucosa* (ALK-Abello, Horsholm, Denmark) or placebo for the three-year course of therapy.

The number of included patients was based on a power calculation that took into account the expected effect size, the standard deviation of the outcomes, and the ordinal variable for the comparative study. The randomization procedure with random selection relied on the use of computer-generated numbers by using a flip-coin generator (Excel 2018, Microsoft Corporation) (Figure 1).

#### 2.13.2. Treatment

The patients were randomly selected to receive Alutard SQ *Betula verrucosa* 20,000 AUM/ml (ALK-Abello, Horsholm, Denmark) or a placebo (Figure 1). The recruitment period was limited to two months (January-February). Alutard SQ is a depot extract containing water-soluble allergen protein with aluminium hydroxide as an adjuvant. The therapy was administered as a perennial therapy using the following regimen: 1 dose of 0.1 ml 100 (SQ-U/ml), 2 doses of 0.1 ml 1000 (SQ-U/ml), 3 doses of 0.1 ml 10,000 (SQ-U/ml), 4 doses of 0.4 ml 10,000 (SQ-U/ml), 5 doses of 0.1 ml 100,000 (SQ-U/ml), 6 doses of 0.2 ml 100,000 (SQ-U/ml), 7 doses of 0.4 ml 100,000 (SQ-U/ml), 8 doses of 0.8 ml 100,000 (SQ-U/ml), and 9 doses of 1.0 ml 100,000 (SQ-U/ml) every week, followed by 1.0 ml 100,000 (SQ-U/ml) every four weeks for three years (2015-2017).

One milliliter of Alutard SQ contains about 12.4 *μ*g of Bet v1. Patients received on average 382 *μ*g of Bet v 1 (range: 282-482 *μ*g) during active treatment for all 36 months of the study.

For study-blinding purposes, all patients received the same volume and the same number of injections. The staff and patients remained study-blind treatment until the investigation database was locked. The placebo as a solution of sterile aluminium hydroxide was packed in the same type of unidentified boxes with identification number only. All key codes were locked by an independent coordinator, who did not participate in the study.

18 patients in the study group and 16 in the placebo completed the study.

#### 2.13.3. Outcomes

The primary outcome measurement was the mean changes in the combined symptom and medication scores (CSMSs) over the birch pollen season after three years of AIT or placebo treatment compared to the baseline before treatment (in 2014). The birch pollen season was defined as the period from 25 March to 20 May when birch spores are detected in the air in Poland. The patients recorded their symptom severity in a daily diary during the birch pollen season by scoring the following areas: nasal itching, sneezing, runny, and blockage, and also, ocular itching, cough, and dyspnea were recorded on a common one visual analogue scale (VAS) with a continuous scale from 0 cm (no symptoms) to 10 cm (very severe symptoms). The final results were the mean of results for individual symptoms.

Rescue medication was provided, and its use was recorded based on the following scoring: one point per spray for Azelastine nasal spray, eye drops (Levocabastine), or per 5 mg levocetirizine tablet, two points per puff per nostril for mometasone furoate nasal spray, and three points per prednisolone 10 mg tablet or salbutamol use on-demand 1-4 inhalation. This combined symptom-medication score was calculated as a sum of the symptom score and medication score and was monitored daily with the use of the diary.

The second outcome measurement was the changes in bronchial reactivity in the methacholine test and response to bronchial provocation tests to birch allergen before and after treatment.

The quality of life, safety assessment, and monitoring of allergen-specific IgE and IgG_4_ were observed against Bet v 1 in the serum at the start of AIT and after every 12 months of a three-year course of therapy.

The local reactions were assessed 30 min after injection and measured in cm. The systemic reactions were graded according to the EAACI criterion [18].

#### 2.13.4. Quality of Life (QoL)

The patients' QoL was evaluated with the RQLQ score for adults using questionnaires every birch pollen season during the study period [19].

#### 2.13.5. Pollen Counts

The local grass pollen counts from March to May were determined using a volumetric pollen trap (Burkard, Scientific Ltd., Uxbridge, UK).

The statistical analysis was performed using Statistica version 8.12 (SoftPol, Cracow, Poland). Nonparametric tests were used because the data were not normally distributed. The Wilcoxon test was used to analyze differences between groups. The chi-square test was used to analyze the differences between nonparametric variables. The ANOVA test was used to analyze the CSMSs. The percentage reduction was calculated as the difference in the area under the curve (AUC). Differences were considered significant at *p* < 0.05.

The study was approved by the local ethics committee of the Medical University of Silesia in Poland (34211). All patients signed an informed consent form. The trial was registered in ClinicalTrials.gov under the Protocol Record NCT 03157505.

## 3. Results

The characteristics of the group are presented in Table 1. 21 (58%) patients presented also symptoms of cough and sometimes other asthmatic symptoms despite rhinitis during the birch pollen season.

### 3.1. The Primary Endpoints

**Figure 2:**
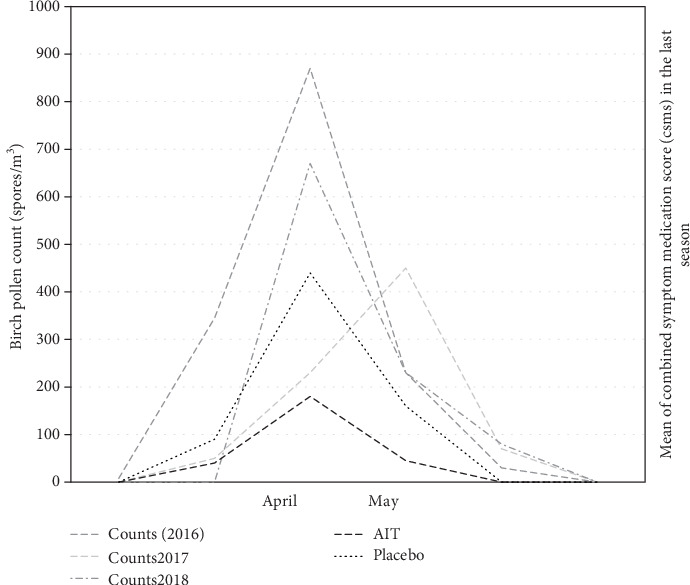
Birch spore counts (2016, 2017, and 2018) and the weekly average of the CSMSs recorded by patients in the active and placebo groups during the last pollen season (2018) after three years of AIT.

The mean CSMSs of three years of AIT were significantly decreased in the active group from 5.88 (range: 4.11-9.01) to 1.98 (range: 1.22-4.51; *p* < 0.05). The detailed data regarding the SMSs, symptom scores, and medication scores prior to the AIT and after two years of treatment are shown in Table 2. The data of birch pollen counts are presented in Figure 2.

### 3.2. Bronchial and Nasal Reactivity

**Figure 3:**
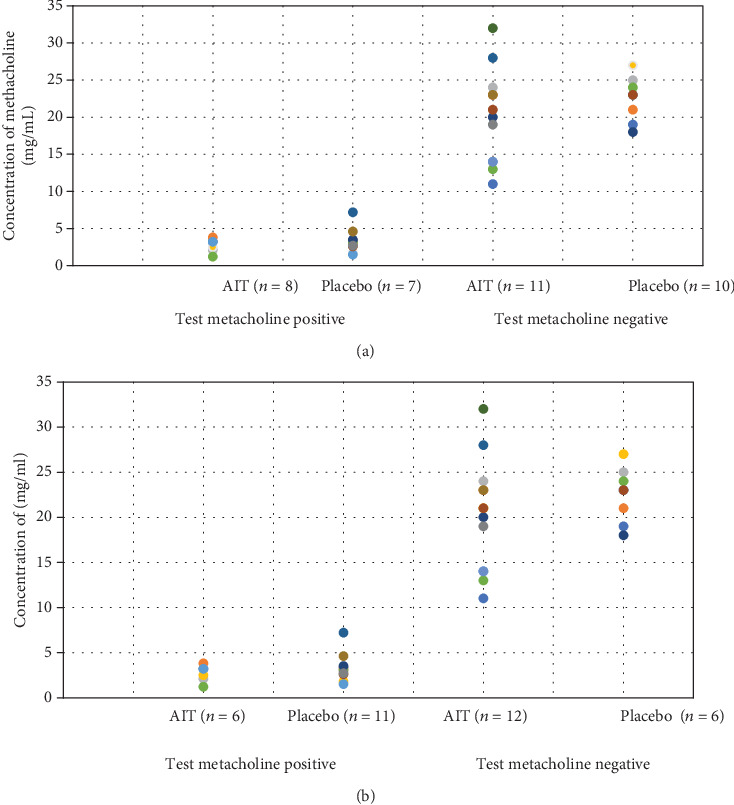
Methacholine tests in studied patients at baseline (a) and after AIT (b).

**Figure 4:**
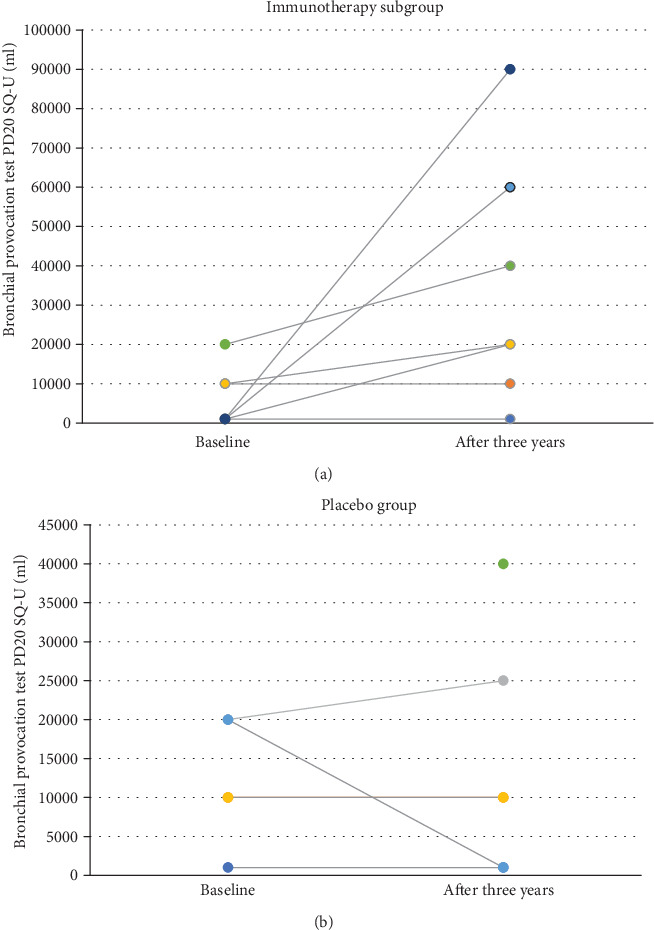
Changes in allergen bronchial provocation tests in the active and placebo groups. In the active group, a significant decrease in bronchial reactivity to birch allergen concentration after allergen immunotherapy was observed (*p* = 0.03). No similar trends in the placebo group were noticed. In one patient, conversion to the positive bronchial reaction was confirmed after three years of observation.

At the start of the study, 18 (50%) included patients presented cough or other asthmatic symptoms despite LAR in the birch pollen season. 16 of them had positive results of methacholine tests, and 11 of them had confirmed the positive bronchial challenge to birch allergen. Bronchial asthma was not diagnosed in any one of them. After AIT, a significant increase in the number of patients with positive methacholine tests in the placebo group was noticed after being observed for three years (*p* = 0.04). At the same time, the significant decrease of bronchial responsiveness to birch allergen in 5 from 7 patients after AIT was confirmed (*p* = 0.03). There are no significant similar changes in the placebo group. The results are presented in Figures 3(a), 3(b), 4(a), and 4(b). There were no significant changes in the results of a bronchial reversibility test in all studied patients during the whole observation.

At baseline, all patients have had positive NPT to birch and the mean concentration of 6250 ± 1200 SQ-U/ml allergen during 15-20 min. After three years of AIT, there was a significant increase of toleration for birch allergen up to 45000 ± 2500 SQ-U/ml (*p* = 0.02) during repeated nasal challenges. There were no similar trends in the placebo group [the mean concentration of allergen: 8000 ± 2000 SQ-U/ml (*p* = 0.31)].

In 6 patients after AIT, there was a correlation between decreases in reactivity for different concentrations of birch allergen in NPT and bronchial challenge (*R* = 0.8, *p* < 0.05).

### 3.3. Secondary Endpoints

#### 3.3.1. Quality of Life (QoL)

The RQLQ results showed a mean baseline level of 1.84 (95% CI: 1.53-1.97) in the studied patients. The mean final result was significantly decreased to 1.45 (95% CI: 1.32-1.62) in the active group after three years of AIT therapy (*p* = 0.03) and not significantly changed in placebo: 1.77 (95% CI: 1.56-1.92).

#### 3.3.2. Allergen-Specific IgE and IgG_4_ Measurements

The serum allergen-specific IgE against Bet v1 appeared in the active group between four and six months after initiation of the AIT (maximum mean level: 14.1 ± 7.02 IU/ml), decreased during the next 6 months, and was undetectable during the remaining treatment time. In the placebo group, the serum-specific IgE remained undetectable throughout the study. Serum IgG4 to bet v 1 increased gradually after 6 months of AIT in the active group to 0.94 ± 0.21 mgA/ml. In the placebo group, the serum specific IgG_4_ Bet v 1 was detectable throughout the study at a constant low level (0.21-0.38 mgA/ml).

#### 3.3.3. Safety

No systemic anaphylactic reactions occurred in either group during the AIT therapy. Erythema or wheals < 5 cm were observed after 101 (21.8%) of all injections, and wheals > 5 cm were observed after 28 (9.5%) of all injections in the active group. No adverse reactions were observed in the placebo group.

## 4. Discussion

The obtained results may indicate that allergen immunotherapy can be an effective and safe treatment method in patients with local allergic rhinitis and with confirmed IgE reaction dependent on birch allergen. The basic parameters of the effectiveness of such treatment were met: CSMS, symptom score, and medication score show significant decreases after three-year course of AIT in comparison to placebo. Despite persistent doubts about the role of IgE mechanisms in local allergic rhinitis, the effectiveness of AIT seems to be a further evidence of the importance of this mechanism. Similar good efficacy regarding AIT in local allergic rhinitis has been confirmed in a few other studies [11, 12, 20]. Patients with LAR and allergies to house dust mites or grass pollen had good effects of such treatment, and this treatment was safe, as in the presented work [11, 12]. Appropriate, restrictive patient qualifications may determine the expected good treatment effectiveness [12, 20]. A positive provocation test with an allergen that is suspected of causing symptoms, a negative diagnosis of systemic allergy, determines the final diagnosis. Also, the authors decided to qualify only those patients whose nasal IgE fraction was screened after allergen challenge. This study increased the chances of confirming the dependent IgE reaction and excluded patients with a lack thereof. However, this tool is difficult to use in everyday clinical practice. Some authors emphasize the value of IgE-specific nasal assay and use this method as a supplement to intranasal provocation, but it is not currently a requirement to diagnose local allergic rhinitis [10, 20, 21]. Other parameters such as the quality of life and transient increase in serum IgE and constant increase in sIgG_4_ were similar to other works and proved the effectiveness of AIT in this disease.

An important phenomenon observed at work was the coexistence of similar asthma symptoms in some patients. They were not previously diagnosed for asthma but presented asthma-like symptoms during the birch pollen season. These symptoms were underestimated because the main symptom was coughing and less often shortness of breath and only for a short period of birch pollination.

The obtained data indicated that in some patients the diagnosis of asthma is possible, as evidenced by a positive methacholine test and positive bronchial challenge to birch allergen. There are doubts that the result of bronchial reversibility tests was no collateral in all patients of this subgroup; however, these breathing maneuvers could be less sensitive than others in such cases. On the other hand, these patients achieved improvement after the occasional use of salbutamol during the pollen season despite antihistamine drugs. Also, in this group of patients, the decrease of PEFR was observed during the birch pollen season (data was not presented).

Unfortunately, the placebo group did not receive significant improvement after only symptomatic treatment. It is worth paying attention to this group, in which the number of patients with positive methacholine tests increased. This may be evidence that in some patients the natural course of LAR leads to asthma which may have a local character.

Despite the limitations of the study and the lack of bronchial lavage, positive provocation tests with birch allergen and a decrease in bronchial reactivity to this allergen in studied patients after AIT may be evidence of an IgE-dependent mechanism of these asthmatic reactions. A similar study but with different allergens was presented by Campo et al. [22].

Based on available data and results of the present work, it is possible that in some groups of patients with the diagnosis of local allergic rhinitis, bronchial asthma can be concomitant. Unfortunately, an attempt to simply prove that the symptoms of such asthma are local and allergic is difficult and requires further investigation. In particular, it is difficult to distinguish between year-round, nonspecific bronchial hyperresponsiveness without typical asthma features and local allergic asthma. The attempt to transfer the model of immune responses in local allergic rhinitis to the bronchial area is currently only a hypothesis and requires research at the molecular level.

There are some limitations in this study: a comparatively small group of studied patients, especially with asthmatic symptoms and lack of bronchial lavage in this subgroup during bronchial procedures.

## 5. Conclusion

Allergen immunotherapy to birch can be useful and safe in patients with local allergic rhinitis and also with concomitant asthmatic symptoms during the pollen season.

The presence of a positive bronchial reaction to birch allergen in some of the analyzed patients with typical symptoms could indicate local allergic asthma. Further studies are needed.

## Figures and Tables

**Table 1:** Characteristics of the study patients.

	Active (*n* = 19)	Placebo (*n* = 17)	*p* value
Age (yrs)	27.5.±6.8	25.3 ± 8.3	0.48
Female (%)	11 (58)	12 (70)	0.07
Duration of rhinitis (yrs)	3.9 ± 1.7	4.1 ± 2.2	0.32
Urban (%)	13 (68)	11 (64)	0.82
Mean symptom score in the basement during the birch pollen season	3.81 ± 1.29	3.29 ± 1.51	0.49
Number of patients with the presence of cough and other asthmatic symptoms during birch pollen season (%)	12 (63)	9 (53)	0.52
(i) Cough	12 (63)	9 (53)	
(ii) Wheezing	9 (47)	7 (41)	
(iii) Dyspnea	6 (32)	7 (41)	
(iv) Shortness of breath	8 (42)	5 (29)	
(v) Chest tightness	5 (47)	6 (35)	
A positive result of bronchial reversibility test (%)	4 (21)	3 (18)	0.29
Specific nasal IgE to birch pollen in nasal lavage (kU/L) after NPT	2.17 ± 0.82	1.92 ± 0.68	0.43

NPT: nasal provocation test.

**Table 2:** Combined symptom medication score (CSMS), symptom score, and medication score comparisons for the active and placebo patients at baseline and after AIT.

Parameter	Baseline	End of AIT	*p* value
CSMS			
*Active*	5.86 (3.79-9.34)	1.98 (1.30-3.1)	0.003
*Placebo*	5.94 (3.82-9.21)	4.98 (3.62-5.67)	0.14
Symptom score			
*Active*	3.4 (2.74-6.19)	1.17 (0.81-1.58)	0.002
*Placebo*	3.7 (2.21-5.87)	3.39 (1.19-4.05)	0.34
Medication score			
*Active*	2.03 (2.94-4.03)	0.45 (0.11-1.01)	0.04
*Placebo*	2.51 (1.98-4.31)	2.18 (1.33-3.76)	0.39

CSMS: combined symptom and medication score; the range is given in brackets.

## Data Availability

All data used to support the findings of this study, including patient records, may be released upon request from the Clinical Department of Internal Disease, Dermatology and Allergology (email: sekretariat.dermatologia@klinika-zabrze.med.pl).
